# Supplementary material for: ABCB1 and ABCG2 Control Brain Accumulation and Intestinal Disposition of the Novel ROS1/TRK/ALK Inhibitor Repotrectinib, While OATP1A/1B, ABCG2, and CYP3A Limit Its Oral Availability
Source: Pharmaceutics. 2021 Oct 21;13(11):1761. doi: 10.3390/pharmaceutics13111761 (PMC8619046; doi:10.3390/pharmaceutics13111761)
Supplement: Supplementary file 1 [file pharmaceutics-13-01761-s001.zip › pharmaceutics-1385018-supplementary.pdf]

pharmaceutics

MDPI

Supplementary Materials: ABCB1 and ABCG2 Control Brain Accumulation and Intestinal Disposition of the Novel ROS1/TRK/ALK Inhibitor Repotrectinib, while OATP1A/1B, ABCG2, and CYP3A Limit Its Oral Availability

Wenlong Li, Rolf W. Sparidans, Maria C. Lebre[1], Jos H. Beijnen and Alfred H. Schinkel

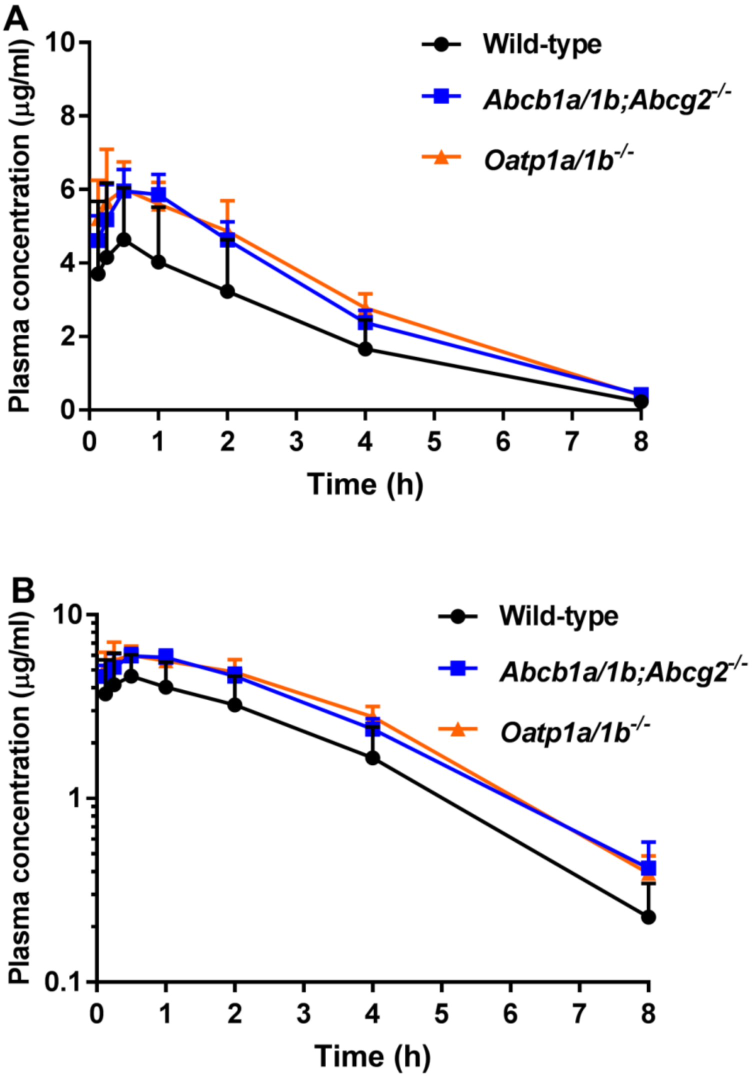

Figure S1. Plasma concentration-time curves (**A**) and semi-log plot of plasma concentration-time curves (**B**) of repotrectinib in female wild-type, *Abcb1a/1b;Abcg2*$^{-/-}$, and *Oatp1a/1b*$^{-/-}$ mice over 8 h after oral administration of 10 mg/kg repotrectinib (n = 6).

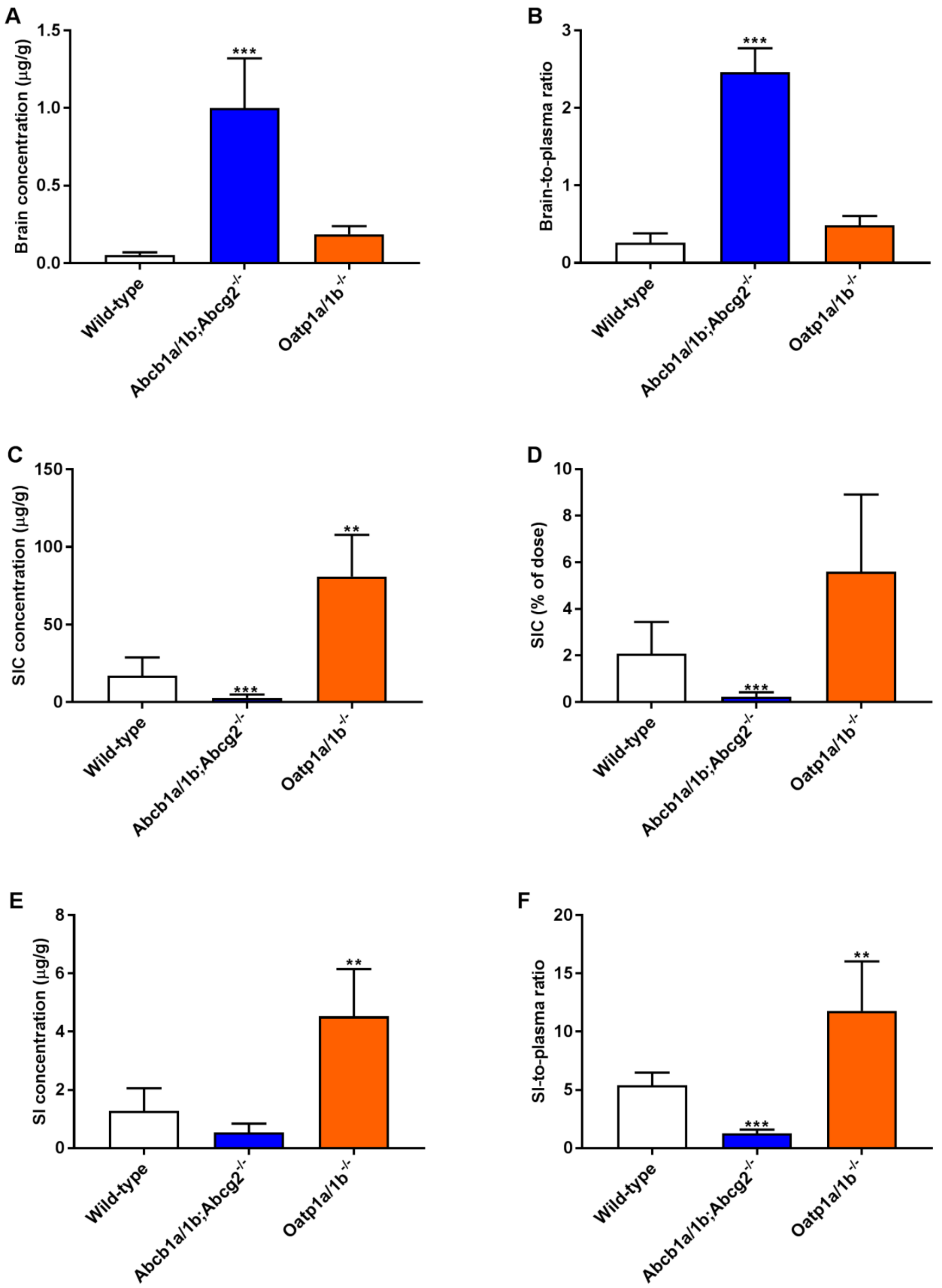

Figure S2. Brain, small intestinal content (SIC) and small intestinal tissue (SI) concentrations (**A, C, E**), and brain- and SI-to-plasma ratios (**B, F**), and SIC as percentage of dose (**D**) of repotrectinib in female wild-type, *Abcb1a/1b;Abcg2*$^{-/-}$, and *Oatp1a/1b*$^{-/-}$ mice 8 h after oral administration of 10 mg/kg repotrectinib. SIC (% of dose), drug percentage of dose in small intestinal content expressed as total repotrectinib in SIC divided by total drug administered to the mouse. Data are presented as mean ± S.D. (n = 6). *, $P < 0.05$; **, $P < 0.01$; ***, $P < 0.001$ compared to wild-type mice.

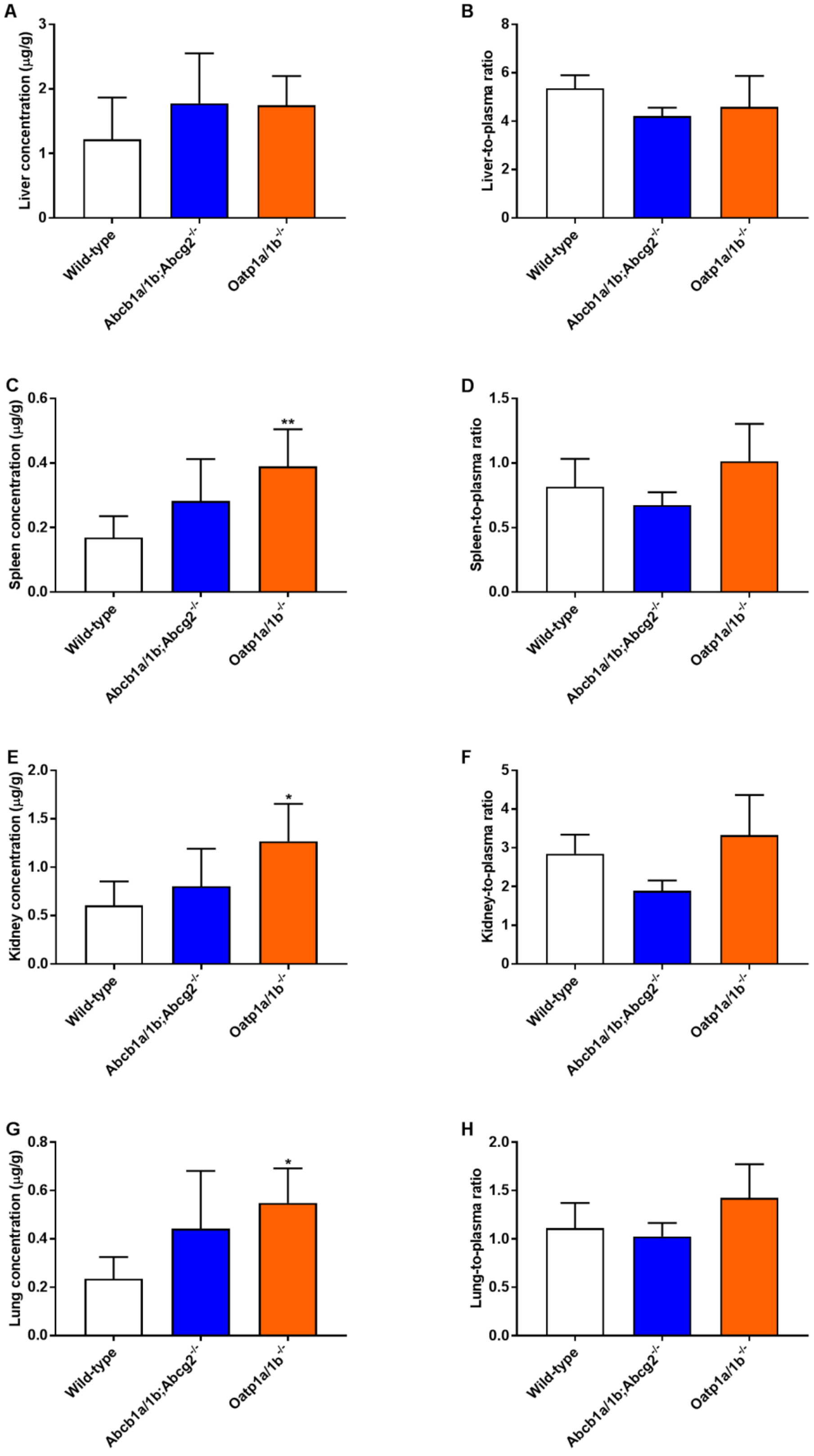

Figure S3. Liver, spleen, kidney and lung concentrations (**A, C, E, G**) and tissue-to-plasma ratios (**B, D, F, H**) of repotrectinib in female wild-type, *Abcb1a/1b;Abcg2*$^{-/-}$, and *Oatp1a/1b*$^{-/-}$ mice 8 h after oral administration of 10 mg/kg repotrectinib. Data are presented as mean ± S.D. (n = 6). *, $P < 0.05$; **, $P < 0.01$; ***, $P < 0.001$ compared to wild-type mice.

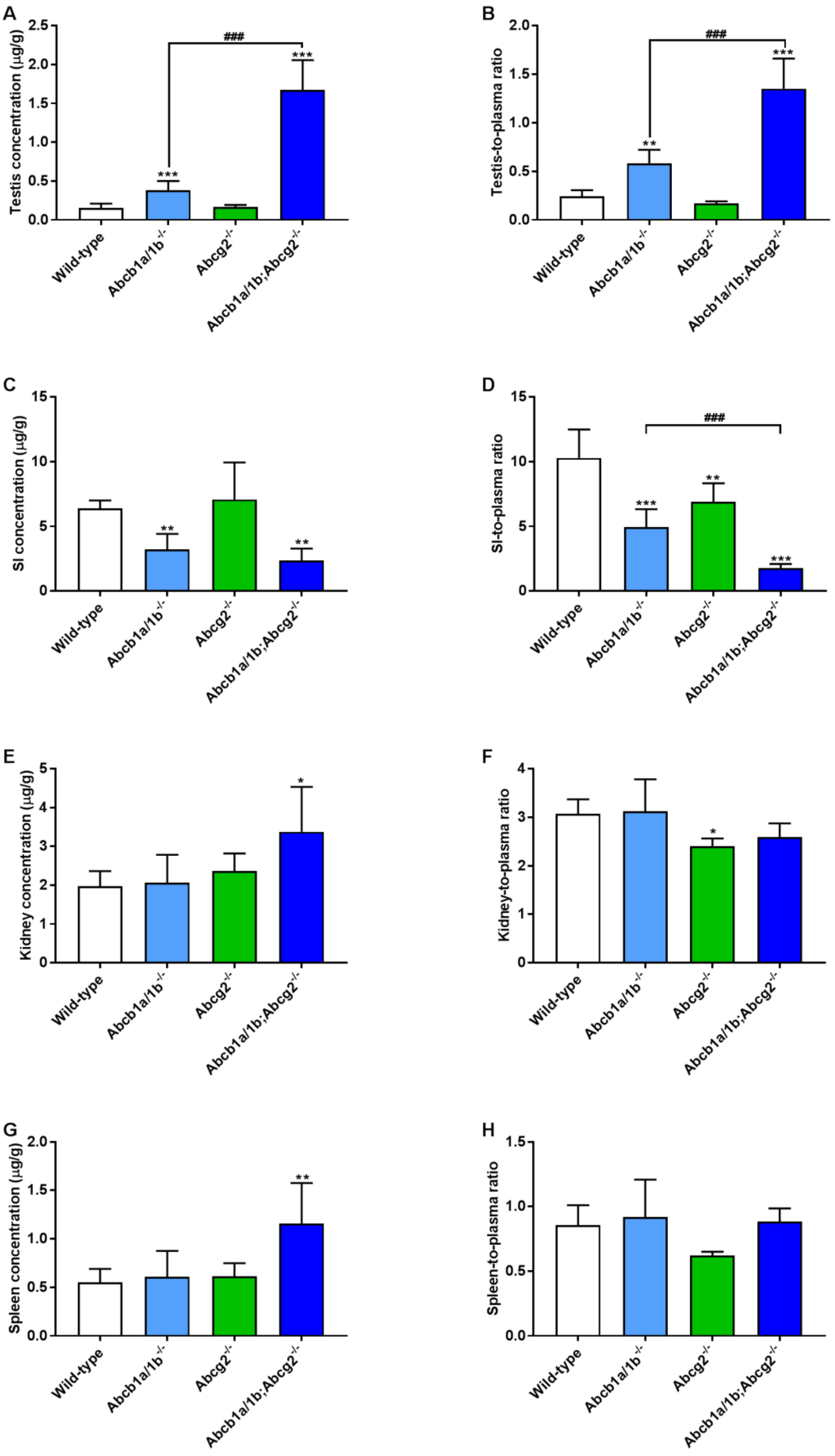

Figure S4. Organ concentrations (**A, C, E, G**) and organ-to-plasma ratios (**B, D, F, H**) of repotrectinib in male wild-type, *Abcb1a/1b*$^{-/-}$, *Abcg2*$^{-/-}$, and *Abcb1a/1b;Abcg2*$^{-/-}$ mice 4 h after oral administration of 10 mg/kg repotrectinib. Data are presented as mean ± S.D. (n = 6-7). *, $P < 0.05$; **, $P < 0.01$; ***, $P < 0.001$ compared to wild-type mice. $^{\#}$, $P < 0.05$; $^{\#\#}$, $P < 0.01$; $^{\#\#\#}$, $P < 0.001$ comparing *Abcb1a/1b;Abcg2*$^{-/-}$ mice to *Abcb1a/1b*$^{-/-}$ mice.

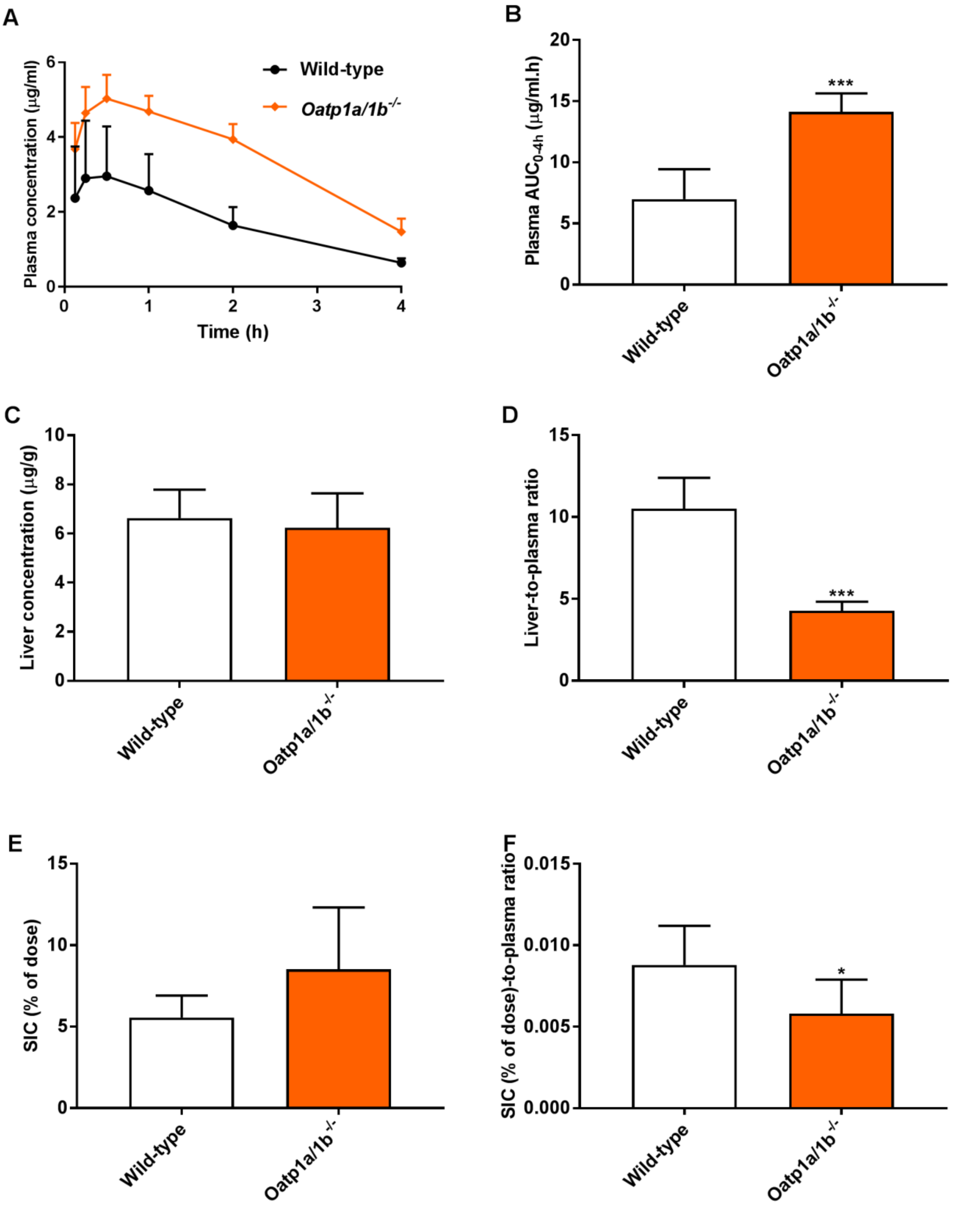

Figure S5. Plasma concentration-time curves (**A**), plasma $AUC_{0\text{-}4h}$ (**B**), liver concentrations (**C**), and liver-to-plasma ratios (**D**), SIC (% of dose) (**E**), and SIC (% of dose)-to-plasma ratios (**F**) of repotrectinib in male wild-type and *Oatp1a/1b*$^{-/-}$ mice 4 h after oral administration of 10 mg/kg repotrectinib. Data are presented as mean ± S.D. (n = 6-7). *, $P < 0.05$; **, $P < 0.01$; ***, $P < 0.001$ compared to wild-type mice.

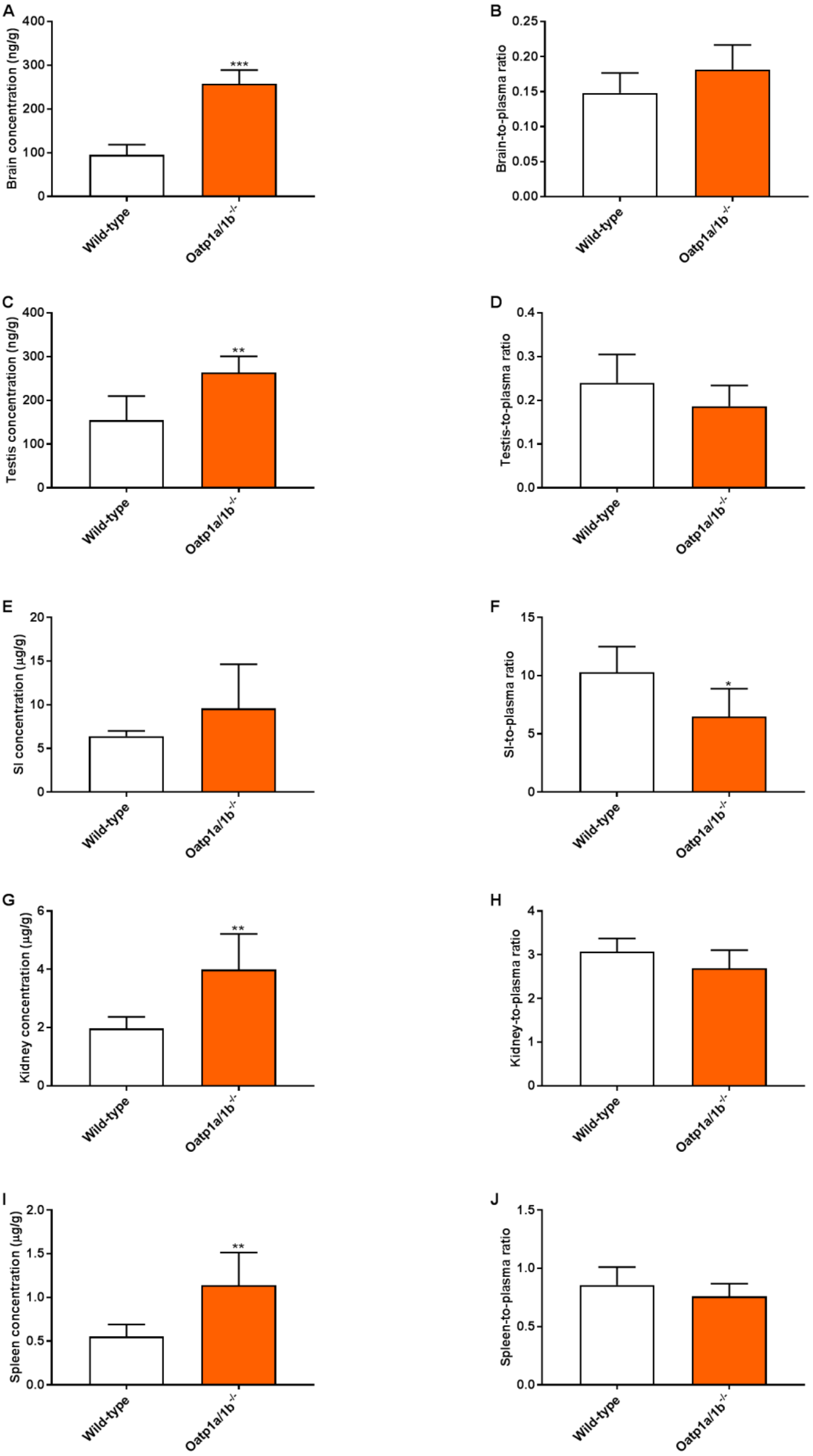

Figure S6. Organ concentrations (**A, C, E, G, I**) and tissue-to-plasma ratios (**B, D, F, H, J**) of repotrectinib in male wild-type and *Oatp1a/1b*$^{-/-}$ mice 4 h after oral administration of 10 mg/kg repotrectinib. Data are presented as mean ± S.D. (n = 6-7). *, $P < 0.05$; **, $P < 0.01$; ***, $P < 0.001$ compared to wild-type mice.

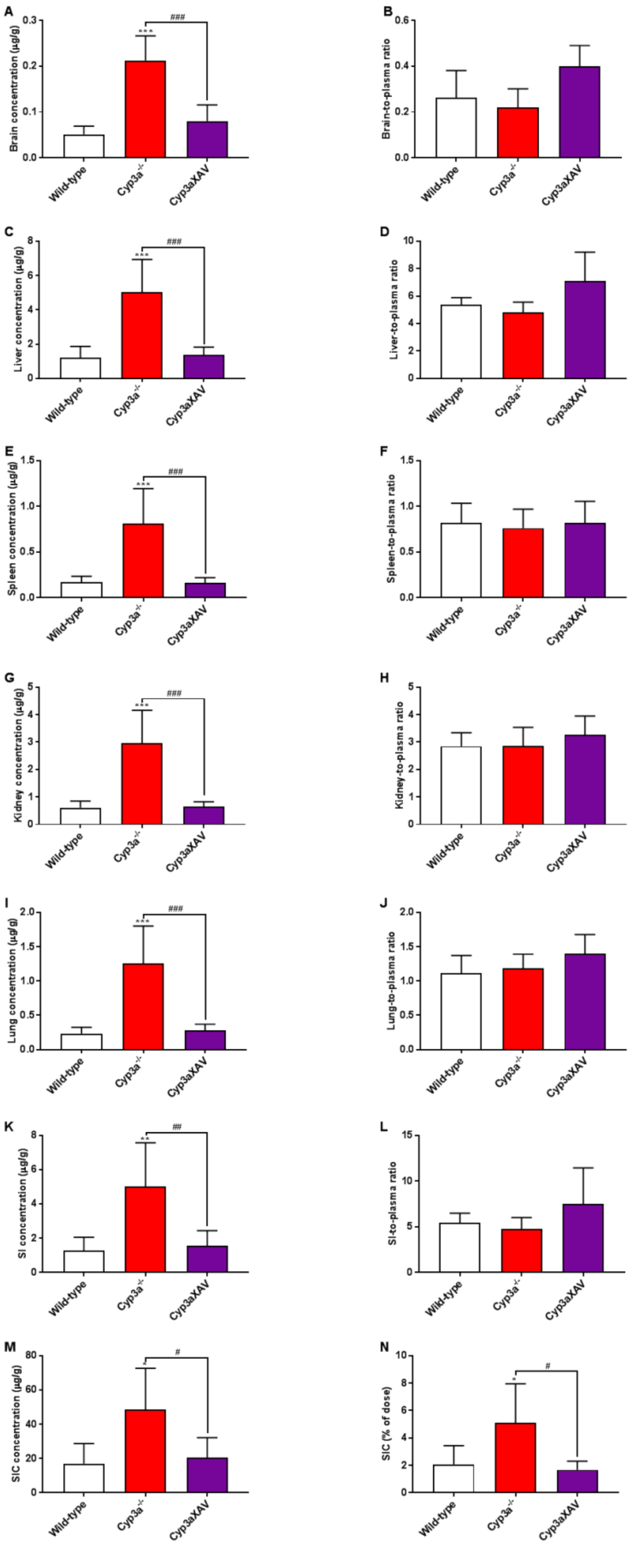

Figure S7. Organ concentrations (**A, C, E, G, I, K**), organ-to-plasma ratios (**B, D, F, H, J, L**), small intestinal content (SIC) concentrations (**M**), and SIC (% of dose) (**N**) of repotrectinib in female wild-type, *Cyp3a*$^{-/-}$, and Cyp3aXAV mice 8 h after oral administration of 10 mg/kg repotrectinib. Data are presented as mean ± S.D. (n = 6). *, *P* < 0.05; **, *P* < 0.01; ***, *P* < 0.001 compared to wild-type mice. $^{\#}$, *P* < 0.05; $^{\#\#}$, *P* < 0.01; $^{\#\#\#}$, *P* < 0.001 comparing Cyp3aXAV to *Cyp3a*$^{-/-}$ mice.

Table S1. Plasma, brain, liver, and small intestine pharmacokinetic parameters of repotrectinib in female wild-type, *Abcb1a/1b;Abcg2*$^{-/-}$, and *Oatp1a/1b*$^{-/-}$ mice over 8 h after oral administration of 10 mg/kg repotrectinib.

**Parameter**	**Genotype**		
	**Wild-type**	***Abcb1a/1b;Abcg2***$^{-/-}$	***Oatp1a/1b***$^{-/-}$
$AUC_{0\text{-}8h}$, μg/ml·h	16.28 ± 6.35	23.10 ± 2.11*	24.60 ± 3.27*
Fold change $AUC_{0\text{-}8h}$	1.00	1.42	1.51
C_{max}, μg/ml	4.80 ± 1.78	6.19 ± 0.44	6.15 ± 0.80
T_{max}, h	0.5 (0.25-1)	0.5 (0.5-1)	0.5 (0.25-1)
C_{brain}, ng/g	51.2 ± 17.8	1000 ± 320.4***	186.2 ± 51.4
Fold increase C_{brain}	1.00	19.5	3.64
Brain-to-plasma ratio	0.26 ± 0.12	2.46 ± 0.31***	0.48 ± 0.12
Fold increase ratio	1.00	9.46	1.85
C_{liver}, μg/ml	1.22 ± 0.65	1.78 ± 0.77	1.75 ± 0.45
Fold increase C_{liver}	1.00	1.50	1.43
Liver-to-plasma ratio	5.36 ± 0.54	4.21 ± 0.35	4.59 ± 1.28
Fold increase ratio	1.00	0.79	0.86
SIC (% of dose)	2.08 ± 1.35	0.22 ± 0.20***	5.59 ± 3.32
Fold change	1.00	0.11	2.69
SIC (% of dose)-to-plasma ratio·10^{-3}	8.87 ± 1.83	0.48 ± 0.43***	14.5 ± 8.9
Fold change	1.00	0.054	1.63

Data are given as mean ± S.D. (n = 6). $AUC_{0\text{-}8h}$, area under the plasma concentration-time curve; C_{max}, maximum concentration in plasma; T_{max}, median time point of maximum plasma concentration; C_{brain}, brain concentration; SIC, small intestinal content. SIC (% of dose), drug percentage of dose in small intestinal content expressed as total repotrectinib in SIC divided by total drug administered to the mouse. *, $P < 0.05$; **, $P < 0.01$; ***, $P < 0.001$ compared to wild-type mice.

Table S2. Plasma, brain, liver, and small intestine pharmacokinetic parameters of repotrectinib in male wild-type and *Oatp1a/1b*$^{-/-}$ mice over 4 h after oral administration of 10 mg/kg repotrectinib.

**Parameter**	**Genotype**	
	**Wild-type**	***Oatp1a/1b***$^{-/-}$
$AUC_{0\text{-}4h}$, μg/ml·h	6.99 ± 2.46	14.12 ± 1.53***
Fold increase $AUC_{0\text{-}4h}$	1.00	2.02
C_{max}, μg/ml	3.08 ± 1.44	5.07 ± 0.59***
T_{max}, h	0.5 (0.25-1)	0.5 (0.25-1)
C_{brain}, ng/g	95.1 ± 23.5	258 ± 31***
Fold increase C_{brain}	1.00	2.71
Brain-to-plasma ratio	0.15 ± 0.03	0.18 ± 0.04
Fold change ratio	1.00	1.20
C_{testis}, ng/g	155 ± 55	264 ± 37**
Fold increase C_{testis}	1.00	1.70
Testis-to-plasma ratio	0.24 ± 0.07	0.19 ± 0.05
Fold change ratio	1.00	0.79
C_{liver} μg/g	6.63 ± 1.16	6.24 ± 1.40
Fold change C_{liver}	1.00	0.94
Liver-to-plasma ratio	10.5 ± 1.9	4.3 ± 0.5***
Fold change ratio	1.00	0.41
SIC (% of dose)	5.56 ± 1.34	8.52 ± 3.80
Fold change SIC (% of dose)	1.00	1.53
SIC (%)-to-plasma ratio *10^{-3}	8.79 ± 2.41	5.81 ± 2.09*
Fold change ratio	1.00	0.66

Data are presented as mean ± S.D. (n = 6-7). $AUC_{0\text{-}4h}$, area under the plasma concentration-time curve; C_{max}, maximum concentration in plasma; T_{max}, median time point of maximum plasma concentration (range for individual mice); $C_{brain/liver/SIC}$, brain/liver/SIC concentration. SIC (% of dose), drug as percentage of dose present in small intestinal content (SIC), which was expressed as total repotrectinib in SIC divided by total drug administered to mouse. *, $P < 0.05$; **, $P < 0.01$; ***, $P < 0.001$ compared to wild-type mice.
